# Establishing a comprehensive host-parasite stable isotope database to unravel trophic relationships

**DOI:** 10.1038/s41597-025-04970-5

**Published:** 2025-04-14

**Authors:** Amandine J. M. Sabadel, Philip Riekenberg, Monica Ayala-Diaz, Mark C. Belk, Jerusha Bennett, Antonio Bode, Sarah J. Bury, Laurent Dabouineau, Josette Delgado, Brittany Finucci, Rita García-Seoane, Luisa Giari, Jessica Henkens, Lonneke L. IJsseldijk, Tijs Joling, Ollie Kerr-Hislop, Colin D. MacLeod, Lauren Meyer, Rona A. R. McGill, Eleonora Negro, Petra Quillfeldt, Cecile Reed, Chloe Roberts, Bahram Sayyaf Dezfuli, Olaf Schmidt, Anthony Sturbois, Andrew D. Suchomel, David W. Thieltges, Carl D. van der Lingen, Marcel T. J. van der Meer, Inés G. Viana, Mark Weston, Trevor J. Willis, Antoine Filion

**Affiliations:** 1 Department of Environmental Science, Auckland University of Technology, Auckland, New Zealand; 2 Zoology Department, University of Otago, Dunedin, New Zealand; 3 National Institute of Water & Atmospheric Research Ltd., Wellington, New Zealand; 4 NIOZ Royal Netherlands Institute for Sea Research, Texel, Netherlands; 5 Biological Sciences Department, University of Alberta, Edmonton, Canada; 6 Department of Biology, Brigham Young University, Provo, Utah USA; 7 Instituto Español de Oceanografía (IEO-CSIC), Centro Oceanográfico de A Coruña, A Coruña, Spain; 8 Catholic University of the West, Guingamp, France; 9 Department of Environmental and Prevention Sciences, University of Ferrara, Ferrara, Italy; 10 College of Science and Engineering, Flinders University, Adelaide, South Australia Australia; 11 Department of Biomolecular Health Sciences, Faculty of Veterinary Medicine, Utrecht University, Utrecht, Netherlands; 12 Biodiversity Research Centre, Department of Zoology, University of British Columbia, Vancouver, Canada; 13 Natural Environment Research Council Life Sciences Mass Spectrometry Facility, Scottish Universities Environmental Research Centre, Rankine Avenue, East Kilbride, UK; 14 Department of Integrative Marine Ecology, Stazione Zoologica Anton Dohrn, Fano Marine Center, viale Adriatico 1-N, Fano, 61032 Italy; 15 Department of Animal Ecology and Systematics, Justus Liebig University Giessen, Giessen, Germany; 16 Department of Biological Sciences, University of Cape Town, Cape Town, South Africa; 17 Department of Life Sciences and Biotechnology, University of Ferrara, Ferrara, Italy; 18 School of Agriculture and Food Science, University College Dublin, Dublin, Ireland; 19 Laboratoire des Sciences de L’Environnement Marin, UMR 6539, Plouzané, France; 20 Vivarmor Nature, Ploufragan, France; 21 Réserve Naturelle Nationale de La Baie de Saint-Brieuc, Hillion, France; 22 Department of Botany and Program in Ecology and Evolution, University of Wyoming, Laramie, Wyoming USA; 23 Groningen Institute for Evolutionary Life Sciences (GELIFES), University of Groningen, P.O. Box 11103, 9700 CC Groningen, The Netherlands; 24 National Biodiversity Future Center, Palermo, Italy

**Keywords:** Stable isotope analysis, Animal behaviour

## Abstract

Over the past decades, stable isotopes have been infrequently used to characterise host-parasite trophic relationships. This is because we have not yet identified consistent patterns in stable isotope values between parasites and their host tissues across species, which are crucial for understanding host-parasite dynamics. To address this, we initiated a worldwide collaboration to establish a unique database of stable isotope values of novel host-parasite pairs, effectively doubling the existing data in published literature. This database includes nitrogen, carbon, and sulphur stable isotope values. We present 3213 stable isotope data entries, representing 586 previously unpublished host-parasite pairs. Additionally, while existing literature was particularly limited in sulphur isotope values, we tripled information on this crucial element. By publishing unreported host-parasite pairs from previously unsampled areas of the world and using appropriate host tissues, our dataset stands unparalleled. We anticipate that end-users will utilise our database to uncover generalisable patterns, deepening our understanding of the complexities of parasite-host relationships and driving future research efforts in stable isotope parasitology.

## Background & Summary

Parasites are ubiquitous within ecosystems, yet their placement within food web representations remain elusive^1–3^. Stable isotope analysis has been extensively applied to describe predator-prey relationships over decades of research, making it a robust and well-tested tool (see Boecklen, *et al*.^4^ for a review). However, the same application in parasite-host settings has been infrequent, despite offering promising insights into these relationships^5^. Stable isotopes have the potential to further clarify host-parasite interactions by:

1. Identifying the feeding strategies of parasites, i.e., are parasites actively or passively feeding on their hosts?
2. Confirming or refuting the classification of a species as ‘parasitic’, i.e., does the species absorb nutrients from its host, or is it dormant?
3. Estimating the amount of biomass rerouted from the host by the parasite
4. Assessing the impact of the parasite on the host’s metabolism and fitness
5. Assessing impacts on host foraging strategies due to parasite infection.

To date, no discernible paradigm for stable isotope fractionation patterns between parasites and hosts have been established, unlike the relatively predictable patterns identified in predator-prey relationships^5–7^, i.e. higher δ^15^N values in predator vs prey. Parasites vary greatly in form and function across all ecosystems (e.g., terrestrial vs. aquatic) and have developed unique evolutionary strategies that influence their stable isotope values, such that the host-parasite stable isotope relationship is more complex than with predators and their prey. Factors such as parasite phylogeny^8^, attachment site (i.e., host gut vs. internal or external tissues)^9^, selective feeding^10,11^, parasite feeding mode (active vs. absorptive)^12,13^, and altered foraging in the host due to behaviour change^14^ or physical impediment^15,16^ all affect the stable isotope values within host-parasite relationships. Thus, despite their potential, stable isotopes have been rarely employed to study host-parasite trophic relationships as their output is often difficult to interpret.

**Fig. 1:**
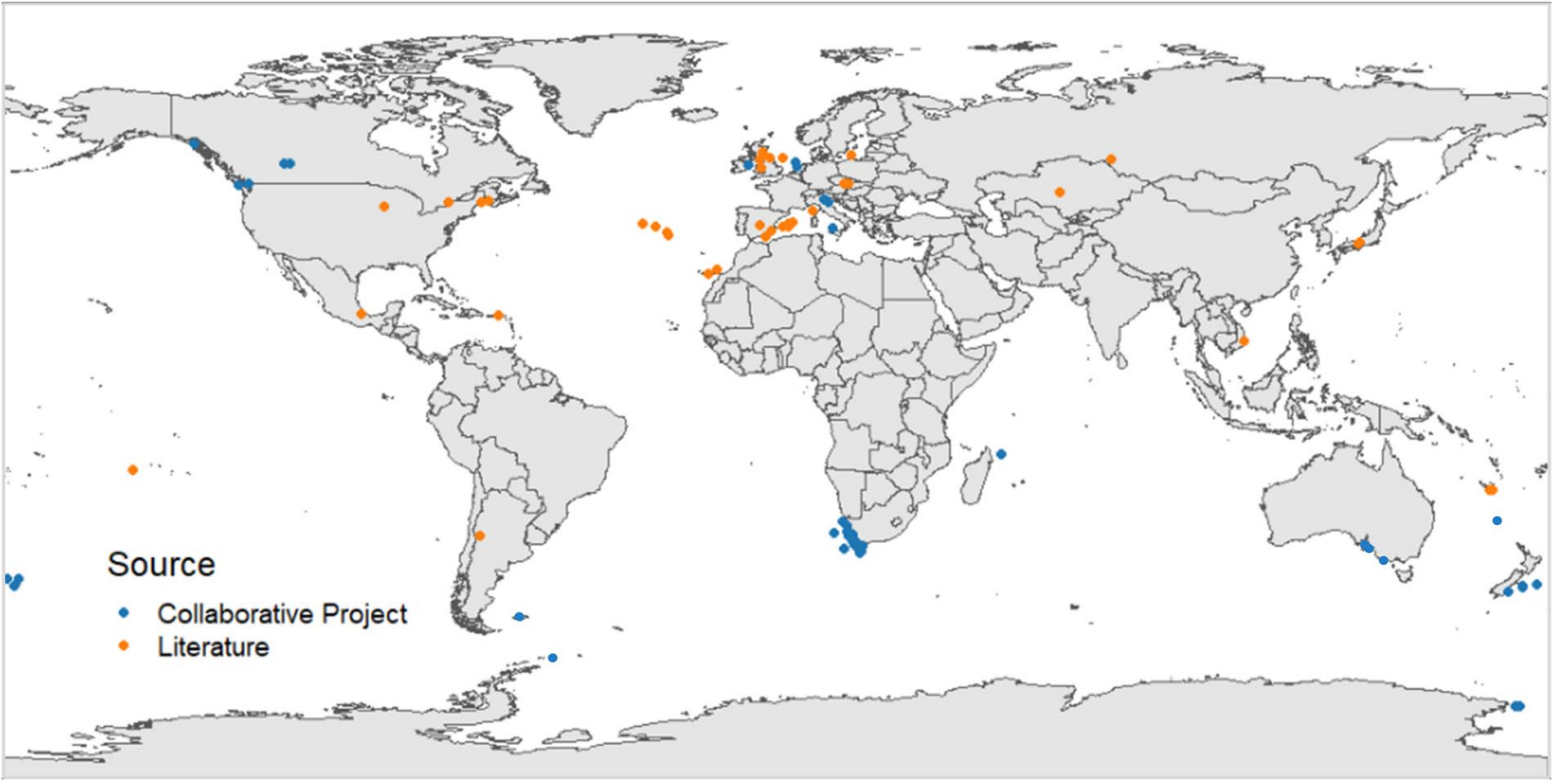
Global distribution of host-parasite pairs. Sampling locations of all host-parasite pairs available from published literature (up to 2022; orange markers) across terrestrial, freshwater, and marine environments, along with our novel, unpublished dataset (blue markers) from this ParaSITE collaborative project. It is important to consider these data globally, as stable isotope baselines vary with geographic location.

In 2019, Thieltges, *et al*.^6^ reviewed the parasitic stable isotope literature up to 2016, with data comprising 101 host-parasite pairs. They noted a significant skew towards fish parasites (over 50%) and highlighted various inconsistencies in sampling protocols, which added an unknown quantity of statistical noise when comparing data. As of the initiation of this ParaSITE (Parasite Stable Isotope Trend Enhancing) project in late 2022, an additional 586 host-parasite pairs had been added to the literature (data not included in this database; orange markers, Fig. 1 for geographical locations), but we remain unable to identify clear, general patterns for trophic discrimination for either carbon or nitrogen stable isotope values between parasite and host, amongst or between parasite groups. Additionally, the published data did not follow systematic protocols, such as comparison against selected host tissue (e.g., material the parasite is directly feeding on), inconsistently applied lipid extraction or adjustment, and often provided inadequate descriptions of host-parasite identifications and pairings, making direct comparison between host-parasite pairs difficult.

**Fig. 2:**
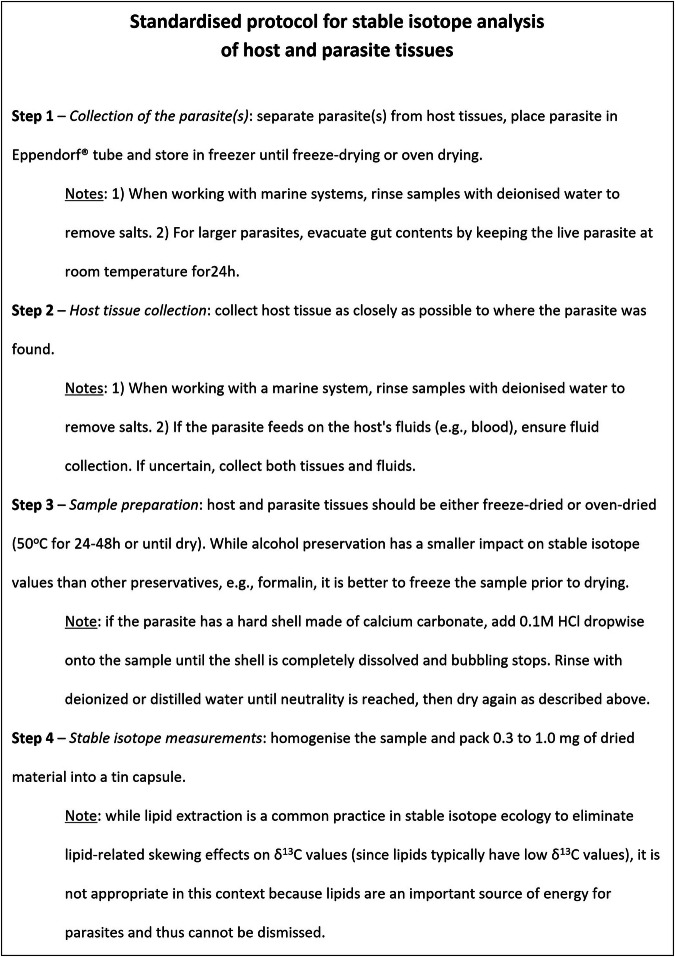
Standardised step-by-step protocol for stable isotope analysis of host-parasite pairs. This template and protocol are designed to ensure consistency and accuracy of reported data and should be applied in future host-parasite stable isotope studies to facilitate reliable host-parasite comparisons and reproducible results.

As such, in 2022 at the 12^th^ International Conference on the Applications of Stable Isotope Techniques to Ecological Studies (IsoEcol 2022, - https://sites.google.com/view/isoecol2020/home?authuser=0), we called upon the interest, curiosity, and generosity of scientists around the world to provide us with new host-parasite pair stable isotope data or samples yet to be measured for δ^13^C, δ^15^N, and δ^34^S values. Two years later, we present the results of this coordinated effort, the first comprehensive database of unpublished host-parasite pairs adhering to the same standardised protocol, described below (Fig. 2), and providing measurements of host tissues on which the parasites selectively feed. Our standardised protocol encompasses sampling methodology, preservation guidelines, and measurement techniques, alongside comprehensive details about the parasite (e.g., feeding mode, taxa) and supporting information (e.g., sampling coordinates, ecosystem type) necessary to provide the framework for revealing host-parasite relationships through meta-analytical approaches.

Overall, since late 2022, we have collected 586 new pairs of host-parasite stable isotope values (3% from terrestrial, 3% from freshwater and 94% from marine environment), which more than doubles the number of already published and available data to date (Fig. 1). Moreover, we tripled the number of host-parasite δ^34^S data from 38 to 107 host-parasite pairs, which until now were only available from two studies^15,17^. The database resulting from the ParaSITE project is composed by fish hosts as the majority (Actinopterygii; 54%), followed by sharks (Chondrichthyes; 18%) and birds (Aves; 18%), gastropods (Gastropoda; 4.7%), mammals (Mammalia; 3.8%), and crustaceans (Malacostraca; 1.6%). The parasites in our dataset span 12 major taxonomic groups, including acanthocephalans, cestodes, nematodes, trematodes, monogeneans, and parasitic crustaceans, among others, highlighting the diversity of host-parasite interactions covered. We acknowledge that this dataset shares the same bias towards fish hosts as the currently published data, but our efforts provide a comprehensive set of new hosts using selected tissue based on parasite feeding styles, explicitly sampled for the purpose of stable isotope parasitology.

This is the first global collaborative effort to establish a baseline dataset for understanding broader trends in stable isotope parasitology, along with a standardised protocol to ensure consistency in future data collection. The present data will not only provide a data set supporting future analyses to uncover host-parasite stable isotope discrimination patterns, but also original stable isotope values for specimens (both parasites and hosts), which are valuable as standalone data supporting work of other researchers looking to incorporate hosts and their parasites into broader food web contexts.

## Methods

### Deciding on the parameters and protocol

Based on gaps identified in the literature, we devised a data-gathering template to collect raw and metadata on both parasites and hosts. This template was developed to fit ISOBANK (https://isobank.tacc.utexas.edu/) standards and includes a wide range of parameters. ISOBANK aims to be a common repository for stable isotope data across disciplines and is therefore a logical and practical archive for this data set. The template and a key that explains each parameter can be found in the Figshare repository under “ParaSITE project_Legend key”. Additionally, we developed a scientifically-based stable isotope sampling protocol (see Fig. 2 below) to ensure that all samples were collected uniformly in order to enable reliable comparisons between stable isotope data within this data set. We encourage scientists to use this protocol for sample collection supporting future studies.

### Acquiring unpublished data

There were two ways of acquiring the stable isotope data for host-parasite pairs included in this data set: (1) Unpublished stable isotope results were sent directly by collaborators using our data-gathering template (see template in the Figshare repository), or (2) Host-parasite pair samples were collected and processed following our standardised protocol (Fig. 2) and were sent directly to New Zealand for stable isotope measurements.

#### Receiving already analysed data

Some collaborators had ready-to-use unpublished data or generously offered to analyse stable isotope values of parasite-host pairs at their preferred analytical facility. In such cases, the data were sent directly via email, using our template, and were checked and curated upon reception. All information on the calibration and reprocessing of these data for each respective laboratory that produced them can be found in the “Technical Validation” section below.

#### Receiving host-parasite pair samples for stable isotope analysis in New Zealand

##### Sampling

Collaborators were encouraged to submit all host-parasite pairs in triplicate, ensuring each pair was unique (e.g., three individual hosts and their matching parasites of the same species), with a targeted amount of 1 mg of dried tissue per sample. Dried samples – see protocol below - were imported into New Zealand using the appropriate import permit from the Zoology Department at the University of Otago (Permit no. 2022080614 issued under The Biosecurity Act, 1993 by the Ministry of Primary Industries, New Zealand).

##### Stable isotope measurements

Stable isotope measurements require between 0.3 to 1.5 mg dry weight per sample, depending on the carbon, nitrogen, and sulphur contents of the tissues. Small parasites of the same species found in the same location within the same host (feeding on the same host tissue) were pooled when necessary to meet the required sample weight. Each sample was pre-weighed into tin capsules on a precision microbalance to 0.001 mg to ensure accurate measurements. We measured carbon (δ^13^C, %C), nitrogen (δ^15^N, %N) and, where possible, sulphur (δ^34^S, %S), stable isotope values and percent element content (See Technical Validation section below for definition of delta values). All stable isotope measurements from samples received in New Zealand were conducted at either Isotrace Ltd, Dunedin, or NIWA, Wellington. Details of the laboratory used for each sample are included in the dataset. All information on the calibration and reprocessing of these data for each respective laboratory that produced the data can be found in the “Technical Validation” section below.

## Data Records

The complete dataset named: “[Article dataset] Establishing a comprehensive host-parasite stable isotope database to unravel trophic relationships”^18^ (10.6084/m9.figshare.28087397) is readily available on Figshare and on ISOBANK^19^ (https://isobank.tacc.utexas.edu/). Our data-gathering template is comprehensive and designed to encompass as much useful information about the parasite-host system as possible. There are 60 required metadata fields, all of which can also be found in the same Figshare repository, as “ParaSITE project_Legend key”. Collaborators were asked to fill in this template as accurately as possible. When some information was not known or was inaccessible, default or “n/a” parameters were selectable.

Note that (1) While this database aims to be as accurate as possible, determination of the exact feeding mechanisms (e.g., active vs absorptive) of all parasites was not always feasible and in these instances the uncertainty is clearly stated in the database. (2) The dataset includes multiple entries for nematode parasites (*Anisakis* spp.) and their respective hosts. Recent work suggests that *Anisakis simplex* do not feed on their paratenic hosts^20^. We retained these data in the database because this finding was true for one fish host species but may not apply to all. (3) Some δ^13^C and δ^15^N data have been previously published^21^, but without the corresponding δ^34^S data, so we included the three isotopes in our database.

For a quick overview of the dataset, we have created a table (Supplement Table S1) that summarises the available parasite-host pairs and their stable isotope values. This table offers a concise reference to the number of pairs available and specifies which isotopes (δ^13^C, δ^15^N, and δ^34^S) were measured for each system.

## Technical Validation

Given that all collaborators followed the protocol for sample handling and preparation, we expect high and comparable quality and reliability in our measurements, eliminating inconsistencies from methodological differences.

This section details the instruments and procedures followed by the laboratories that contributed to the stable isotope measurements in this study. Calibration and reprocessing procedures are key in stable isotope science, as they allow for accurate data comparison.

### For all laboratories

All stable isotope values herein are expressed as parts per thousand (‰) using the formula:

1 $${\rm{\delta }}{\rm{X}}=\left(\frac{{{\rm{R}}}_{{\rm{sample}}}}{{{\rm{R}}}_{{\rm{standard}}}}-1\right)\times 1000$$

Where δX is δ^13^C, δ^15^N, or δ^34^S and R is the respective ^13^C/^12^C, ^15^N/^14^N, or S^34^/S^32^ ratio of the sample or the standard being measured. The standards used to calibrate the δX values were Vienna Peedee Belemnite (VPDB) for carbon, atmospheric N_2_ for nitrogen, or Canyon Diablo troilite (CDT) for sulphur.

No samples were lipid-corrected to ensure an accurate understanding of nutrient flow from host to parasite. Lipid correction can alter stable isotope values and lipids are a crucial energy source for parasites.

### Environmental and ecological stable isotope analytical facility at national institute of water & atmospheric research Ltd. (NIWA), wellington, New Zealand

Stable isotope analyses were carried out on a DELTA V Plus continuous flow isotope ratio mass spectrometer (IRMS) linked to a Flash 2000 elemental analyser (EA) using a MAS 200 R autosampler (Thermo Fisher Scientific, Bremen, Germany) at the NIWA Environmental and Ecological Stable Isotope Facility in Wellington, New Zealand. More detailed methodology is outlined in^22^. Both δ^13^C and δ^15^N values were measured, along with the elemental content of the sample in C (expressed as %C) and N (expressed as %N). Values of δ^13^C and %C were two-point normalised using stable isotope data from the daily analysis of NIST 8573 USGS40 L-glutamic acid (δ^13^C = −26.39 ± 0.09‰ and %C = 40.82) and NIST 8542 IAEA-CH-6 Sucrose (δ^13^C = −10.45 ± 0.07‰ and %C = 42.11), whilst values of δ^15^N and %N were two-point normalised using stable isotope data from the daily analysis of NIST 8573 USGS40 L-glutamic acid (δ^15^N = −4.52 ± 0.12‰ and %N = 9.52) and NIST 8548 IAEA-N2 Ammonium sulphate (δ^15^N = 20.41 ± 0.07‰ and %N = 21.2). Repeat analysis of USGS74 L-Valine #2 and USGS65 Glycine produced data accurate to within 0.07‰ for δ^13^C and 0.05‰ for δ^15^N, and 0.50% and 0.32% for %C and %N respectively. Precision was determined by the repeat analysis of a working laboratory standard DL-Leucine (DL-2-Amino-4-methylpentanoic acid, C_6_H_13_NO_2_, Lot 127H1084, Sigma, Australia) and was ± 0.08‰ for δ^13^C, ±0.07‰ for δ^15^N, ±0.25 for %C, and ±0.16 for %N.

### Isotope ratio mass spectrometry unit, dunedin, New Zealand

Values of δ^13^C, δ^15^N and δ^34^S, along with the elemental compositions of carbon, nitrogen, and sulphur, were measured on an EA Isolink CNSOH coupled with a Delta V Advantage IRMS (Thermo Fisher Scientific, Bremen, Germany). The samples were standardised to international isotope reference materials G01, a mix of USGS40 and IAEA-S1 (δ^15^N = −4.52‰, δ^13^C = −26.39‰ and δ^34^S = −0.30‰) and G02, a mix of USGS41 and IAEA-S2 (δ^15^N = 47.55‰, δ^13^C = 36.55‰ and δ^34^S = 22.62‰). The quality control was conducted by applying an in-house laboratory control material, Keratin Internal Standard (δ^15^N = 8.91‰, δ^13^C = −21.14‰ and δ^34^S = 13.08‰). Instrument precision was 0.05‰ for δ^15^N values, 0.07‰ for δ^13^C and 0.60‰ for δ^34^S.

### Stable isotope laboratory, royal Netherlands institute for Sea research (NIOZ), texel, Netherlands

Samples were analysed with a Elementar BiovisION consisting of an Elementar Vario Isotope Cube elemental analyser connected to an Isoprime VisION isotope ratio mass spectrometer at NIOZ (Texel, Netherlands. Analytical precision for CNS analysis was ±0.1‰ for δ^13^C, ±0.2‰ for δ^15^N, and 0.3‰ for δ^34^S, with samples scaled against certified international refence materials Acetanilide (Arndt Schimmelmann, Indiana University), and IAEA-S2 and S3 for sulphur. Every analytical run includes several other certified standard materials as additional control; EMA-P1 and Casein (Elemental Microanalysis).

### Servicio de apoyo a la investigación, universidade da coruña, coruña, Spain

Stable isotope analyses were carried out on an EA FlashEA1112, coupled via a ConFlo IV interface to a DeltaV Advantage IRMS (all Thermo Fisher Scientific, Bremen, Germany). Standards used include USGS 40 (δ^13^C = −26.39‰, δ^15^N = −4.52‰), USGS41a (δ^13^C = 36.55‰, δ^15^N = 47.55‰), USGS 24 (δ^13^C = −16.049‰), NBS 22 (δ^13^C = −30.031‰), IAEA-N-1 (δ^15^N = 0.4‰), IAEA-N-2 (δ^15^N = 20.3‰), and USGS-25 (δ^15^N = −30.4‰). Every analytical run also included internal standards (acetanilide, n = 10) of known isotopic composition, and samples were measured in triplicate to evaluate precision (±SD) which was ±0.14‰ for δ^13^C and δ^15^N, ±0.89 for %C, and ±0.29 for %N.

### Centre for the isotope sciences - national environmental isotopes facility (neif), scottish universities environmental research centre (SUERC), Glasgow, UK

Samples were analysed using a vario Pyrocube (Elementar Analysensysteme GmbH, Hanau, Germany) coupled to a Delta XP Plus IRMS (Thermo Fisher Scientific, Bremen, Germany). Linearity and drift corrections are made daily using in house lab standards: gel, glygel and alagel laboratory standards (Fluka Gelatine, Alagel is a mixture of 13C-Alanine, and Fluka Gelatine, Glygel is a mixture of Sigma Glycine, Fluka Gelatine and 15N-Alanine). Two laboratory standards were analysed every 10 samples. Four aliquots of USGS40 are run daily as an unknown – analytical precision for 32 USGS40 run over eight experiments containing parasite samples (March 2023-February 2024) was ±0.06‰ for δ^13^C, ±0.16‰ for δ^15^N. Lab standards are corrected monthly relative to USGS40, USGS41, USGS25, IAEA CH6, IAEA N1 and IAEA N2.

### LEMAR, pôle spectrométrie océan, university of bretagne occidentale, brest, France

Stable isotope measurements were conducted using an elemental analyser (EA Flash 2000 from ThermoFisher Scientific) coupled with an IRMS (Delta V Plus from ThermoFisher Scientific) at the stable isotope platform of the Pole Spectrométrie Océan at the University of Bretagne Occidentale (Brest, France). Reference standard used were Vienna-Pee Dee Belemnite for ^13^C and atmospheric N_2_ for ^15^N. Standards used to estimate accuracy included USGS-61 (δ^13^C = −35.07‰, δ^15^N = −2.87‰), USGS-62 (δ^13^C = −14.73‰, δ^15^N = 20.17‰), USGS-63 (δ^13^C = −1.21‰, δ^15^N = 37.83‰). Analytical error was estimated by replicate measurements (n = 11) of: thermos-acetanilide (SD δ^13^C = ± 0.12‰, SD δ^15^N = ± 0.13‰).

### Stable light isotope laboratory, department of archaeology, university of Cape Town, Cape Town, South Africa

Analysis of bulk stable isotope was conducted on a Sartorius M2P (Sartorius AG, Goettingen, Germany) microbalance. Samples were analysed using a Flash 2000 (Thermo Fisher Scientific, Bremen, Germany) organic elemental analyser and the gases directed to a Delta V Plus isotope ratio mass spectrometer via a Conflo IV (Thermo Fisher Scientific, Bremen, Germany) gas control unit. In-house standards used for calibrations were valine, Merck gel and seal bone. The valine is DL-Valine produced by SIGMA which has a δ^15^N of 12.14‰ ± 0.15 and a δ^13^C of −26.80‰ ± 0.14; Merck gel is a proteinaceous gel produced by Merck with a δ^15^N of 7.50‰ ± 0.16 and a δ^13^C of −20.05‰ ± 0.14; and seal bone is crushed bone which was demineralized and dissolved in acid and then reconstituted into a gel standard and has a δ^15^N of 15.84‰ ± 0.13 and a δ^13^C of −11.97‰ ± 0.10. All of these inhouse standards were calibrated with the International Atomic Agency standards, with nitrogen being expressed in terms of its value compared to atmospheric nitrogen, and carbon expressed in terms of its value compared to VPDB.

### Cornell university stable isotope laboratory (coil), cornell university, Cornell, USA

The samples were analysed using a Therma Delta V isotopic ratio mass spectrometer (IRMS) interfaced to a NC2500 elemental analyser. To ensure accuracy and precision of the samples, an in-house standard was analysed after every 10 samples. Based on the result of these samples, values have an error associated with the linearity of 0.21‰ for δ^15^N and 0.08‰ for δ^13^C.

### Flinders analytical, flinders university, adelaide, Australia

Samples were analysed for bulk δ^13^C and δ^15^N content using an Isoprime GC5 Continuous Flow IRMS with vario ISOTOPE cube elemental analyser (Elementar Australia Pty Ltd). Standards used for calibration were ran every ten samples and included L-glutamic acid (USGS40) (δ^13^C = −26.39‰, δ^15^N = −4.52‰), marine collagen peptides (δ^13^C = −20.80‰, δ^15^N = + 5.55‰), and enriched caffeine (δ^13^C = −25.90‰, δ^15^N = + 80.70‰) (Sercon Ltd, UK) Analytical accuracy, evaluated using standards, averaged 0.07‰ ± 0.05‰ for δ^15^N and 0.08‰ ± 0.04‰ for δ^13^C. Analytical precision averaged 0.29‰ ± 0.11‰ for δ^15^N and 0.06‰ ± 0.01‰ for δ^13^C.

## Usage Notes

We hope that these data will encourage and support future stable isotope studies, aiming to identify recognisable patterns that will advance the use of stable isotopes and accelerate the growth of the field of stable isotope parasitology.

## Supplementary information

Supplement

## Data Availability

No coding was necessary to curate this database.

## References

[1] Dobson, A., Lafferty, K. D., Kuris, A. M., Hechinger, R. F. & Jetz, W. Homage to Linnaeus: How many parasites? How many hosts? *Proceedings of the National Academy of Sciences* **105**, 11482–11489, 10.1073/pnas.0803232105 (2008). 10.1073/pnas.0803232105 PMC2556407 18695218

[2] Kuris, A. M. *et al*. Ecosystem energetic implications of parasite and free-living biomass in three estuaries. *Nature* **454**10.1038/nature06970 (2008). 10.1038/nature06970 18650923

[3] Marcogliese, D. J. & Cone, D. K. Food webs: a plea for parasites. *Trends in Ecology & Evolution* **12**, 320–325, 10.1016/S0169-5347(97)01080-X (1997). 10.1016/S0169-5347(97)01080-X 21238094

[4] Boecklen, W. J., Yarnes, C. T., Cook, B. A. & James, A. C. On the use of stable isotopes in trophic ecology. *Annual Review of Ecology, Evolution, and Systematics* **42**, 411–440, 10.1146/annurev-ecolsys-102209-144726 (2011).

[5] Sabadel, A. J. M., Stumbo, A. D. & MacLeod, C. D. Stable isotope analysis: a neglected tool for placing parasites in food webs. *Journal of Helminthology* **93**, 1–7, 10.1017/S0022149X17001201 (2019). 29486814 10.1017/S0022149X17001201

[6] Thieltges, D. W., Goedknegt, M. A., O’Dwyer, K., Senior, A. M. & Kamiya, T. Parasites and stable isotopes: a comparative analysis of isotopic discrimination in parasitic trophic interactions. *Oikos*10.1111/oik.06086 (2019).

[7] Lafferty, K. D. *et al*. Parasites in food webs: the ultimate missing links. *Ecology Letters* **11**, 533–546, 10.1111/j.1461-0248.2008.01174.x (2008). 18462196 10.1111/j.1461-0248.2008.01174.x PMC2408649

[8] Riekenberg, P. M. *et al*. Isotopic discrimination in helminths infecting coral reef fishes depends on parasite group, habitat within host, and host stable isotope value. *Scientific Reports* **11**, 4638, 10.1038/s41598-021-84255-0 (2021). 33633261 10.1038/s41598-021-84255-0 PMC7907083

[9] Born-Torrijos, A. *et al*. Parasite effects on host’s trophic and isotopic niches. *Trends in Parasitology* **39**, 749–759, 10.1016/j.pt.2023.06.003 (2023). 37451950 10.1016/j.pt.2023.06.003

[10] Deudero, S., Pinnegar, J. K. & Polunin, N. V. C. Insights into fish host-parasite trophic relationships revealed by stable isotope analysis. *Diseases of Aquatic Organisms* **52**, 77–86, 10.3354/dao052077 (2002). 12517008 10.3354/dao052077

[11] Pinnegar, J. K., Campbell, N. & Polunin, N. V. C. Unusual stable isotope fractionation patterns observed for fish hostparasite trophic relationships. *Journal of Fish Biology* **59**, 494–503, 10.1111/j.1095-8649.2001.tb02355.x (2001).

[12] Sabadel, A. J. M. & MacLeod, C. D. Stable isotopes unravel the feeding mode–trophic position relationship in trematode parasites. *Journal of Animal Ecology* **91**, 484–495, 10.1111/1365-2656.13644 (2022). 34860441 10.1111/1365-2656.13644

[13] Gilbert, B. M. *et al*. You are how you eat: differences in trophic position of two parasite species infecting a single host according to stable isotopes. *Parasitology Research* **119**, 1393–1400, 10.1007/s00436-020-06619-1 (2020). 32030511 10.1007/s00436-020-06619-1 PMC7176597

[14] Welicky, R. L. & Sikkel, P. C. Decreased movement related to parasite infection in a diel migratory coral reef fish. *Behavioral Ecology and Sociobiology* **69**, 1437–1446, 10.1007/s00265-015-1956-3 (2015).

[15] Sabadel, A. J. M., Cresson, P., Finucci, B. & Bennett, J. Unravelling the trophic interaction between a parasitic barnacle (Anelasma squalicola) and its host Southern lanternshark (Etmopterus granulosus) using stable isotopes. *Parasitology* **149**, 1976–1984, 10.1017/S0031182022001299 (2022). 36076261 10.1017/S0031182022001299 PMC10090636

[16] Vivas Muñoz, J. C. *et al*. Eye fluke infection changes diet composition in juvenile European perch (*Perca fluviatilis*). *Sci Rep* **11**, 3440, 10.1038/s41598-021-81568-y (2021). 33564005 10.1038/s41598-021-81568-y PMC7873217

[17] Becker, E. L., Cordes, E. E., Macko, S. A., Lee, R. W. & Fisher, C. R. Using stable isotope compositions of animal tissues to infer trophic interactions in Gulf of Mexico lower slope seep communities. *PLOS ONE* **8**, e74459, 10.1371/journal.pone.0074459 (2013). 24324572 10.1371/journal.pone.0074459 PMC3855623

[18] Sabadel, A. J. M. *et al*. [Article dataset] Establishing a comprehensive host-parasite stable isotope database to unravel trophic relationships. *Figshare*10.6084/m9.figshare.28087397 (2025). 10.1038/s41597-025-04970-5 PMC11997146 40229317

[19] Shipley, O. N. *et al*. Design, development, and implementation of IsoBank: A centralized repository for isotopic data. *PLOS ONE* **19**, e0295662, 10.1371/journal.pone.0295662 (2024). 39240878 10.1371/journal.pone.0295662 PMC11379280

[20] Sabadel, A. *et al*. Just hitching a ride: stable isotopes reveal non-feeding behaviour of *Anisakis simplex* within its host fish. *Journal of Fish Diseases*, e14043 10.1111/jfd.14043 (2024). 10.1111/jfd.14043 39528846

[21] Riekenberg, P. M. *et al*. Stable nitrogen isotope analysis of amino acids as a new tool to clarify complex parasite–host interactions within food webs. *Oikos* **130**, 1650–1664, 10.1111/oik.08450 (2021).

[22] Bury, S. J. *et al*. Southern Ocean humpback whale trophic ecology. I. Combining multiple stable isotope methods elucidates diet, trophic position and foraging areas. *Marine Ecology Progress Series* **734**, 123–155, 10.3354/meps14532 (2024).

