# Supplementary material for: Establishing a comprehensive host-parasite stable isotope database to unravel trophic relationships
Source: Sci Data. 2025 Apr 14;12:623. doi: 10.1038/s41597-025-04970-5 (PMC11997146; doi:10.1038/s41597-025-04970-5)
Supplement: Supplementary file 1 — Supplement [file 41597_2025_4970_MOESM1_ESM.pdf]

Supplement

Table S1. An overview table of all available parasite-host pairs and their corresponding stable isotope values. Ordered by parasite groups.

Parasite Group	Parasite Class	Parasite Species	Host Group	Host Class	Host Species	Number of Pairs Available	Stable Isotope Values Available
Acanthocephala	Palaeacanthocephala	*Polymorphus minutus*	gammarid amphipod	Malacostraca	*Gammarus roeselii*	1	$\delta^{13}C$, $\delta^{15}N$, and $\delta^{34}S$
Acanthocephala	Palaeacanthocephala	*Pomphorhynchus laevis*	gammarid amphipod	Malacostraca	*Echinogammarus stammeri*	3	$\delta^{13}C$, $\delta^{15}N$, and $\delta^{34}S$
Acanthocephala	Palaeacanthocephala	*Pomphorhynchus laevis*	Common barbel	Actinopterygii	*Barbus barbus*	3	$\delta^{13}C$, $\delta^{15}N$, and $\delta^{34}S$
Acanthocephala	Palaeacanthocephala	*Pomphorhynchus laevis*	Channel catfish	Actinopterygii	*Ictalurus punctatus*	6	$\delta^{13}C$, $\delta^{15}N$, and $\delta^{34}S$
Acanthocephala	Eoacanthocephala	*Neoechinorhynchus agilis*	Thinlip mullet	Actinopterygii	*Chelon ramada*	7	$\delta^{13}C$, $\delta^{15}N$, and $\delta^{34}S$
Acanthocephala	Palaeacanthocephala	*Polymorphus minutus*	gammarid amphipod	Malacostraca	*Gammarus roeselii*	1	$\delta^{13}C$, $\delta^{15}N$, and $\delta^{34}S$
Barnacle	Theostraca	*Sacculina carcini*	Green shore crab	Malacostraca	*Carcinus maenas*	3	$\delta^{13}C$, $\delta^{15}N$, $\delta^{34}S$
Barnacle	Thecostraca	*Anelasma squalicola*	Southern lanternshark	Elasmobranchii	*Etmopterus granulosus*	2	$\delta^{13}C$, $\delta^{15}N$, $\delta^{34}S$
Cestode	Cestoda	*Bothriocephalus scorpii*	Kelp greenling	Actinopterygii	*Hexagrammos decagrammus*	1	$\delta^{13}C$ and $\delta^{15}N$

Cestode	Cestoda	*Bothriocephalus scorpii*	Pacific cod	Actinopterygii	*Gadus macrocephalus*	1	$\delta^{13}C$ and $\delta^{15}N$
Cestode	Cestoda	*Diphyllobothriidae gen.* spp.	Antarctic silverfish	Actinopterygii	*Pleuragramma antarctica*	29	$\delta^{13}C$, $\delta^{15}N$, and $\delta^{34}S$
Cestode	Cestoda	*Diphyllobothrium sp.*	Harbour porpoise	Mammalia	*Phocoena phocoena*	1	$\delta^{13}C$, $\delta^{15}N$, and $\delta^{34}S$
Cestode	Cestoda	*Ormatiobothrium* sp.	Rig shark	Elasmobranchii	*Mustelus lenticulatus*	4	$\delta^{13}C$, $\delta^{15}N$, and $\delta^{34}S$
Cestode	Cestoda	*Tetrabothrius* sp.	White capped albatross	Aves	*Thalassarche steadi*	1	$\delta^{13}C$, $\delta^{15}N$, and $\delta^{34}S$
Copepod	Copepoda	*Lepeophtheirus salmonis*	Pink salmon	Actinopterygii	*Oncorhynchus gorbuscha*	8	$\delta^{13}C$ and $\delta^{15}N$
Copepod	Copepoda	*Lepeophtheirus salmonis*	Chum salmon	Actinopterygii	*Oncorhynchus keta*	3	$\delta^{13}C$ and $\delta^{15}N$
Copepod	Hexanauplia	*Pseudocycnus appendiculatus*	Yellowfin tuna	Actinopterygii	*Thunnus albacares*	4	$\delta^{13}C$ and $\delta^{15}N$
Copepod	Hexanauplia	*Euryphorus brachypterus*	Yellowfin tuna	Actinopterygii	*Thunnus albacares*	7	$\delta^{13}C$ and $\delta^{15}N$
Copepod	Hexanauplia	*Pseudocycnus appendiculatus*	Albacore	Actinopterygii	*Thunnus alalunga*	16	$\delta^{13}C$ and $\delta^{15}N$
Copepod	Hexanauplia	*Euryphorus brachypterus*	Albacore	Actinopterygii	*Thunnus alalunga*	13	$\delta^{13}C$ and $\delta^{15}N$
Copepod	Hexanauplia	*Euryphorus brachypterus*	Bigeye tuna	Actinopterygii	*Thunnus obesus*	21	$\delta^{13}C$ and $\delta^{15}N$
Copepod	Hexanauplia	*Pseudocycnus appendiculatus*	Bigeye tuna	Actinopterygii	*Thunnus obesus*	2	$\delta^{13}C$ and $\delta^{15}N$

Copepod	Hexanauplia	*Euryphorus brachypterus*	Southern bluefin tuna	Actinopterygii	*Thunnus maccoyii*	6	$\delta^{13}C$ and $\delta^{15}N$
Copepod	Hexanauplia	*Hatschekia conifera*	Atlantic pomfret	Actinopterygii	*Brama brama*	6	$\delta^{13}C$ and $\delta^{15}N$
Copepod	Hexanauplia	*Brachiella elegans*	Yellowtail amberjack	Actinopterygii	*Seriola lalandi*	4	$\delta^{13}C$ and $\delta^{15}N$
Copepod	Hexanauplia	*Parabrachiella* sp.	Yellowtail amberjack	Actinopterygii	*Seriola lalandi*	8	$\delta^{13}C$ and $\delta^{15}N$
Copepod	Hexanauplia	*Brachiella* sp.	Yellowtail amberjack	Actinopterygii	*Seriola lalandi*	2	$\delta^{13}C$ and $\delta^{15}N$
Copepod	Hexanauplia	*Caligus bonito*	Atlantic bonito	Actinopterygii	*Sarda sarda*	14	$\delta^{13}C$ and $\delta^{15}N$
Copepod	Hexanauplia	*Chondracanthus colligens*	Kingklip	Actinopterygii	*Genypterus capensis*	1	$\delta^{13}C$ and $\delta^{15}N$
Copepod	Hexanauplia	*Cardiodectes bellottii*	Hector's lanternfish	Actinopterygii	*Lampanyctodes hectoris*	11	$\delta^{13}C$ and $\delta^{15}N$
Copepod	Hexanauplia	*Hatschekia conifera*	Snoek, barracouta	Actinopterygii	*Thyrsites atun*	2	$\delta^{13}C$ and $\delta^{15}N$
Copepod	Hexanauplia	*Caligus* sp.	Snoek, barracouta	Actinopterygii	*Thyrsites atun*	5	$\delta^{13}C$ and $\delta^{15}N$
Copepod	Hexanauplia	*Clavellisa Ilishae*	Pilchard	Actinopterygii	*Sardinops sagax*	3	$\delta^{13}C$ and $\delta^{15}N$
Copepod	Hexanauplia	*Nemesis lamna lamna*	Shortfin mako	Elasmobranchii	*Isurus oxyrinchus*	7	$\delta^{13}C$ and $\delta^{15}N$
Copepod	Hexanauplia	*Kroyeria carchariaeglauci*	Blue shark	Elasmobranchii	*Prionace glauca*	4	$\delta^{13}C$ and $\delta^{15}N$

Copepod	Copepoda	*Caligidae sp.*	Southern bluefin tuna	Actinopterygii	Thunnus maccoyii	7	$\delta^{13}C$ and $\delta^{15}N$
Copepod	Copepoda	*Caligidae sp.*	White shark	Elasmobranchii	*Carcharodon carcharias*	1	$\delta^{13}C$ and $\delta^{15}N$
Copepod	Copepoda	*Caligidae sp.*	Port Jackson shark	Elasmobranchii	*Heterodontus portusjacksoni*	2	$\delta^{13}C$ and $\delta^{15}N$
Isopod	Malacostraca	*Rocinela belliceps*	Red Irish Lord	Actinopterygii	*Hemilepidotus hemilepidotus*	1	$\delta^{13}C$ and $\delta^{15}N$
Isopod	Malacostraca	*Gnathia* sp.	East Atlantic peacock wrasse	Actinopterygii	*Symphodus tinca*	13	$\delta^{13}C$ and $\delta^{15}N$
Isopod	Malacostraca	*Gnathia* sp.	Painted comber	Actinopterygii	*Serranus scriba*	69	$\delta^{13}C$ and $\delta^{15}N$
Isopod	Malacostraca	*Gnathia sp.*	Tiger shark	Elasmobranchii	*Galeocerdo cuvier*	1	$\delta^{13}C$ and $\delta^{15}N$
Leech	Clitellata	*Notostomum cyclostomum*	Pacific cod	Actinopterygii	*Gadus macrocephalus*	1	$\delta^{13}C$ and $\delta^{15}N$
Leech	Clitellata	*Beringobdella rectangulata*	Pacific cod	Actinopterygii	*Gadus macrocephalus*	1	$\delta^{13}C$ and $\delta^{15}N$
Leech	Clitellata	*Trachelobdella* sp.	Mediterranean damselfish	Actinopterygii	*Chromis chromis*	2	$\delta^{13}C$ and $\delta^{15}N$
Leech	Clitellata	*Trachelobdella* sp.	Mediterranean rainbow wrasse	Actinopterygii	*Coris julis*	2	$\delta^{13}C$ and $\delta^{15}N$
Leech	Clitellata	*Trachelobdella* sp.	East Atlantic peacock wrasse	Actinopterygii	*Symphodus tinca*	1	$\delta^{13}C$ and $\delta^{15}N$
Leech	Clitellata	*Trachelobdella* sp.	Painted comber	Actinopterygii	*Serranus scriba*	1	$\delta^{13}C$ and $\delta^{15}N$

Leech	Clitellata	*Piscicolidae sp.*	Port Jackson shark	Elasmobranchii	*Heterodontus portusjacksoni*	36	$\delta^{13}C$ and $\delta^{15}N$
Leech	Clitellata	*Piscicolidae sp.*	Southern fiddler ray	Elasmobranchii	*Trygonorrhina dumerilii*	5	$\delta^{13}C$ and $\delta^{15}N$
Lice	Malacostraca	*Isocyamus deltobranchium*	Harbour porpoise	Mammalia	*Phocoena phocoena*	1	$\delta^{13}C$, $\delta^{15}N$, $\delta^{34}S$
Monogenean	Monogenea	*Entobdella stenolepis*	Halibut	Actinopterygii	*Hippoglossus stenolepis*	2	$\delta^{13}C$ and $\delta^{15}N$
Monogenean	Monogenea	*Monogenean* sp. 1	Yellowfin tuna	Actinopterygii	*Thunnus albacares*	1	$\delta^{13}C$ and $\delta^{15}N$
Monogenean	Monogenea	*Nasicola klawei*	Albacore	Actinopterygii	*Thunnus alalunga*	4	$\delta^{13}C$ and $\delta^{15}N$
Monogenean	Monogenea	*Hexostoma* sp.	Bigeye tuna	Actinopterygii	*Thunnus obesus*	15	$\delta^{13}C$ and $\delta^{15}N$
Monogenean	Monogenea	*Tristoma adcoccineum*	Swordfish	Actinopterygii	*Xiphias gladius*	4	$\delta^{13}C$ and $\delta^{15}N$
Monogenean	Monogenea	*Anthocotyle merluccii*	Cape hake	Actinopterygii	*Merluccius capensis*	2	$\delta^{13}C$ and $\delta^{15}N$
Monogenean	Monogenea	*Diclidophoridae*	Snoek, barracouta	Actinopterygii	*Thyrsites atun*	1	$\delta^{13}C$ and $\delta^{15}N$
Monogenean	Monogenea	*Gastrocotyle trachuri*	Cape horse mackerel	Actinopterygii	*Trachurus capensis*	5	$\delta^{13}C$ and $\delta^{15}N$
Monogenean	Monogenea	*Monogenean* sp. 1	Yellowfin tuna	Actinopterygii	*Thunnus albacares*	1	$\delta^{13}C$ and $\delta^{15}N$
Nematode	Chromadorea	*Anisakis simplex*	Halibut	Actinopterygii	*Hippoglossus stenolepis*	18	$\delta^{13}C$ and $\delta^{15}N$

Nematode	Chromadorea	*Pseudoterranova decipiens*	Halibut	Actinopterygii	*Hippoglossus stenolepis*	12	$\delta^{13}C$ and $\delta^{15}N$
Nematode	Chromadorea	*Hysterothylacium sp.*	Halibut	Actinopterygii	*Hippoglossus stenolepis*	3	$\delta^{13}C$ and $\delta^{15}N$
Nematode	Chromadorea	*Anisakis simplex*	Pacific cod	Actinopterygii	*Gadus macrocephalus*	8	$\delta^{13}C$ and $\delta^{15}N$
Nematode	Chromadorea	*Pseudoterranova decipiens*	Pacific cod	Actinopterygii	*Gadus macrocephalus*	11	$\delta^{13}C$ and $\delta^{15}N$
Nematode	Chromadorea	*Hysterothylacium sp.*	Pacific cod	Actinopterygii	*Gadus macrocephalus*	2	$\delta^{13}C$ and $\delta^{15}N$
Nematode	Chromadorea	*Anisakis simplex*	Sablefish	Actinopterygii	*Anoplopoma fimbria*	10	$\delta^{13}C$ and $\delta^{15}N$
Nematode	Chromadorea	*Pseudoterranova decipiens*	Sablefish	Actinopterygii	*Anoplopoma fimbria*	1	$\delta^{13}C$ and $\delta^{15}N$
Nematode	Chromadorea	*Anisakis simplex*	Arrowtooth flounder	Actinopterygii	*Atheresthes stomias*	1	$\delta^{13}C$ and $\delta^{15}N$
Nematode	Chromadorea	*Anisakis simplex*	Great sculpin	Actinopterygii	*Myoxocephalus polyacanthocephalus*	1	$\delta^{13}C$ and $\delta^{15}N$
Nematode	Chromadorea	*Anisakis simplex*	Pink Salmon	Actinopterygii	*Oncorhynchus gorbuscha*	1	$\delta^{13}C$ and $\delta^{15}N$
Nematode	Chromadorea	*Anisakis simplex*	Chum salmon	Actinopterygii	*Oncorhynchus keta*	1	$\delta^{13}C$ and $\delta^{15}N$
Nematode	Chromadorea	*Anisakis simplex*	Atka mackerel	Actinopterygii	*Pleurogrammus monopterygius*	2	$\delta^{13}C$ and $\delta^{15}N$
Nematode	Chromadorea	*Anisakis simplex*	Searcher	Actinopterygii	*Bathymaster signatus*	1	$\delta^{13}C$ and $\delta^{15}N$

Nematode	Chromadorea	*Hysterothylacium sp.*	Searcher	Actinopterygii	*Bathymaster signatus*	1	$\delta^{13}C$ and $\delta^{15}N$
Nematode	Chromadorea	*Anisakis* sp.	Antarctic silverfish	Actinopterygii	*Pleuragramma antarctica*	5	$\delta^{13}C$, $\delta^{15}N$, and $\delta^{34}S$
Nematode	Chromadorea	*Anisakis simplex* sensu lato	White capped albatross	Aves	*Thalassarche steadi*	1	$\delta^{13}C$, $\delta^{15}N$, and $\delta^{34}S$
Nematode	Chromadorea	*Anisakis simplex* sensu lato	White chinned petrel	Aves	*Procellaria aequinoctialis*	1	$\delta^{13}C$, $\delta^{15}N$, and $\delta^{34}S$
Tick	Arachnida	*Ixodes ricinus*	Mongolian gerbil	Mammalia	*Meriones unguiculatus*	12	$\delta^{13}C$ and $\delta^{15}N$
Tick	Arachnida	*Ixodes ricinus*	European rabbit	Mammalia	*Oryctolagus cuniculus*	6	$\delta^{13}C$ and $\delta^{15}N$
Trematode	Trematoda	*Meiogimnofalus minutus*	Common cockle	Bivalvia	*Cerastoderma edule*	6	$\delta^{13}C$ and $\delta^{15}N$
Trematode	Trematoda	*Crytocotyle concava*	Common goby	Actinopterygii	*Pomatoschistus microps*	5	$\delta^{13}C$ and $\delta^{15}N$
Trematode	Trematoda	*Campula oblonga*	Harbour porpoise	Mammalia	*Phocoena phocoena*	1	$\delta^{13}C$, $\delta^{15}N$, $\delta^{34}S$
Trematode	Trematoda	*Cryptocotyle lingua*	Common periwinkle	Gastropoda	*Littorina littorea*	4	$\delta^{13}C$, $\delta^{15}N$, $\delta^{34}S$
Trematode	Trematoda	*Cardiocephaloides ovicorpus*	Triplefin	Actinopterygii	Tripterygiidae	1	$\delta^{13}C$, $\delta^{15}N$, and $\delta^{34}S$
Trematode	Trematoda	*Himasthla leptosoma*	Gastropod	Gastropoda	*Littorina scutulata*	5	$\delta^{13}C$, $\delta^{15}N$, and $\delta^{34}S$
Trematode	Trematoda	*Himasthla quissetensis*	Gastropod	Gastropoda	*Ilyanassa obsoleta*	11	$\delta^{13}C$, $\delta^{15}N$, and $\delta^{34}S$

Trematode	Trematoda	*Austrobilharzia variglandis*	Gastropod	Gastropoda	*Ilyanassa obsoleta*	3	$\delta^{13}C$, $\delta^{15}N$, and $\delta^{34}S$
Trematode	Trematoda	Plagiorchis sp.	Gastropod	Gastropoda	*Stagnicola elodes*	3	$\delta^{13}C$, $\delta^{15}N$, and $\delta^{34}S$
Flea larvae	Insecta	Parapsyllus sp.	Prion	Aves	*Pachyptila belcheri*	6	$\delta^{13}C$ and $\delta^{15}N$
Flea	Insecta	Parapsyllus sp.	Prion	Aves	*Pachyptila belcheri*	9	$\delta^{13}C$ and $\delta^{15}N$
Mallophage	Insecta	Mallophaga sp.	Prion	Aves	*Pachyptila belcheri*	2	$\delta^{13}C$ and $\delta^{15}N$
Tick	Arachnida	*Ixodes uriae*	Penguin	Aves	*Eudyptes chrysocome*	7	$\delta^{13}C$ and $\delta^{15}N$
Flea	Insecta	Parapsyllus sp.	Penguin	Aves	*Eudyptes chrysocome*	4	$\delta^{13}C$ and $\delta^{15}N$
Mallophage	Insecta	Mallophaga sp.	Penguin	Aves	*Eudyptes chrysocome*	1	$\delta^{13}C$ and $\delta^{15}N$
